# Factors Influencing Men’s Experiences and Engagement with the Rugby Fans in Training—New Zealand Pilot Trial: A Healthy Lifestyle Intervention for Men

**DOI:** 10.3390/healthcare9121737

**Published:** 2021-12-16

**Authors:** Elaine Anne Hargreaves, Samantha Marsh, Ralph Maddison

**Affiliations:** 1 School of Physical Education, Sport and Exercise Sciences, University of Otago, Dunedin 9016, New Zealand; 2 National Institute for Health Innovation, School of Population Health, The University of Auckland, Auckland 1010, New Zealand; sam.marsh@auckland.ac.nz (S.M.); ralph.maddison@deakin.edu.au (R.M.); 3 Institute for Physical Activity and Nutrition (IPAN), School of Exercise and Nutrition Sciences, Deakin University, Melbourne, VIC 3125, Australia

**Keywords:** men’s health, overweight/obesity, behaviour change, physical activity, healthy eating, qualitative

## Abstract

Health promotion programs designed specifically to support men to improve their lifestyle behaviours are required to improve men’s health. This study explored factors that influenced men’s experiences of, and engagement with, the Rugby Fans in Training—New Zealand pilot trial, a professional sport-based healthy lifestyle intervention for overweight men. Thirty-five men (mean age = 45, SD = 10 years) who completed the 12-week intervention participated in one of eight semi-structured focus groups. Using inductive thematic analysis, five themes represented the meanings in the data. First, a group of like-minded men all in the same boat recognised the importance of being in similar life situations and having similar reasons for joining the programme. Second, the men described the importance of the support and motivation provided by the team atmosphere created through the programme. Third, the motivational coach recognised the characteristics, skills and knowledge of the coach delivering the programme which created engagement with it. Fourth, the education sessions were valued for the knowledge gained and underpinning philosophies that guided them. Finally, the involvement of the rugby franchise influenced commitment to the programme and created initial interest. These results provide evidence for the key components that should be incorporated into the future development of and improvement to healthy lifestyle interventions for men.

## 1. Introduction

New Zealand men have a 4-year lower life expectancy, are more likely to suffer coronary heart disease, Type II diabetes, high cholesterol, have a stroke, and tend to develop certain chronic conditions at an earlier age, than New Zealand women [1,2]. Furthermore, compared with women, the prevalence of being overweight or obese is greater in men (70% vs. 63%) [2]. Reasons for this are multifactorial but poor lifestyle habits are likely contributing factors [3]. For example, men are more likely than women to consume a diet low in fruit and vegetables and have higher alcohol consumption, while 42% do not achieve recommended levels of physical activity [2]. Furthermore, men are repeatedly underrepresented in traditional health promotion interventions aimed at improving lifestyle behaviours and reduce weight [4]. Programmes or initiatives designed to improve health and well-being within the health-care system need to ensure they are gender-responsive and equitable to ensure they address the health needs of men [5]. Here, we describe the experiences of the men who completed the pilot Rugby Fans in Training—New Zealand programme (RuFIT-NZ) [6].

The recent emergence of gender-sensitised (in terms of context, content and delivery) healthy lifestyle programmes for men, situated within the context of professional sport, are reversing this trend of male underrepresentation in health promotion programmes, attracting men frequently described as ‘hard to engage’ or the ‘un-reached’ [7,8,9]. Arguably the most rigorously evaluated of these was the Football Fans in Training (FFIT) programme, a 12-week gender-sensitised weight management intervention for overweight men aged between 35 and 65 years delivered through Scottish Premier League football clubs [10]. Adaptations of FFIT have since been delivered through European football [11], Canadian Ice Hockey [8,12], Australian Rules Football [13], and UK Rugby Union [6]. These programmes were underpinned by key theory based behaviour change techniques shown to be effective in improving physical activity and healthy eating [14]. These programmes have demonstrated significant short- [6,8,13,15] and longer-term [9,11,12,16] changes in health outcomes (e.g., weight loss, improved blood pressure) and lifestyle behaviour change (e.g., eating more fruit and vegetables, increased physical activity). Less information has been provided about why these programmes were effective in changing behaviour. For public health practice and future intervention development, it is imperative that research focuses on the specific components of the programmes that promote engagement and influence behaviour change.

To date, understanding men’s experiences with participating in these programmes has been limited to process evaluations of FFIT [17,18], the Canadian Hockey FIT [19] and Hat Trick programs [20], and Aussie-FIT [21]. These four studies identified key factors underpinning participant satisfaction: (1) the relationships developed, and support provided, from the ‘like-minded’ men participating, (2) positive interactions with the coaches delivering the programme, (3) the content of the programme, including how it was delivered (FFIT, Aussie-FIT), the ice hockey themed content (Hat Trick), and the importance of small, manageable, realistic behaviour changes (Hat Trick, Aussie-FIT), and (4) the programme being situated within the sport stadium with close association with the sports club (Hat Trick, FFIT, Aussie-FIT).

The purpose of this study was to understand the ‘active ingredients’ that explain participant experiences of, and engagement with, our pilot RuFIT-NZ trial. The importance of evaluating the mechanisms inherent within an intervention that bring about change has been recognised [22,23]. Furthermore, understanding the context in which the intervention is situated is critical to this evaluation because an intervention may have different effects in different contexts (e.g., a football to rugby union context and UK to New Zealand cultural context), despite implementation being relatively similar [22] and so can add to extant literature. Identifying the important characteristics of intervention effectiveness during the feasibility and pilot phase provides a unique opportunity to further refine and adapt the intervention for subsequent delivery in a full trial [23].

## 2. Materials and Methods

### 2.1. Setting and Context

RuFIT-NZ was a lifestyle intervention focused on improving physical activity and dietary behaviours, delivered through professional rugby union franchises. Men eligible to participate were those who were aged between 25–65 years, overweight (BMI ≥ 25 kg/m^2^ [24]), physically inactive (accumulate < 150 min of moderate intensity or <75 min of vigorous intensity physical activity per week, [25]), and could safely participate in physical activity (physical consent was required for those who were identified at risk after completing the Physical Activity Readiness Questionnaire [26]). Complete details of the protocol and feasibility and pilot outcomes have been reported elsewhere [6]. In brief, the 12-week RuFIT-NZ intervention was delivered as weekly sessions by a RuFIT-NZ coach. Each weekly session comprised (1) an individually tailored, group-based, exercise training session that progressed in challenge across time, and (2) a workshop-based education component focused on supporting men to make changes to nutrition behaviours and alcohol consumption, physical activity, sedentary behaviour, and sleep. It was delivered using evidence-based behaviour change techniques (e.g., goal setting, social support, self-monitoring) [14], recognised guidelines for weight management [25,27] and culturally tailored for New Zealand men. Participants were randomised into the intervention and wait-list control groups. The wait-list control group received no lifestyle behaviour information or physical activity intervention, and received the RuFIT-NZ intervention after the 12-week follow-up period.

### 2.2. Participants

All 44 men who completed the RuFIT-NZ programme at the Dunedin based Highlanders franchise were invited by email to participate in a focus group discussion discussing their experiences of the programme. The men were drawn from the intervention (*n* = 20) and wait-list control (*n* = 24) groups who had completed the same programme just 12 weeks apart. All 44 men replied that they were interested in participating and scheduled a time to attend a group discussion. However, only 35 of those 44 men actually attended. The remaining nine did not show up to their scheduled focus group. We offered these men the opportunity to participate in a subsequent session but they were unable to attend. Participants were aged between 27 and 63 years (mean = 44.5, SD = 10.0 years), were largely of New Zealand European ethnicity (91%), 89% worked full-time, 51% were educated to high school level and 40% had university-level qualifications, participants varied substantially in income level (*n* = 3 < $30,000, *n* = 18 $51–90,000, *n* = 5 > 90), 74% were married. Ethical approval for the study was obtained through the University of Auckland Human Participants Ethics Committee (ref: 014067) and participant consent was obtained. Data were collected in August 2016.

### 2.3. Focus Group Discussion

Focus group discussion was chosen as the method through which to generate the data. This approach is appropriate when the purpose of the group is to gather a range of opinions, and factors that influence those opinions, from people with characteristics in common that relate to the topic of interest [28]; in this case, the factors that influenced participants’ experiences of the RuFIT-NZ programme. The moderator (first author) managed the group dynamics to ensure all participants contributed to the discussion and maintained focus on the research objectives and discussion [29]. Discussion between participants was facilitated allowing individuals to respond and react to what others were saying, creating confirmation that those experiences also existed for them, and allowing the men to voice experiences that they may not have thought about without prompting from their peers [30]. The facilitator also adopted an investigative role, by asking specific questions and engaging in dialogue with participants where necessary to probe for depth, detail, and clarity in participant contributions [30].

### 2.4. Procedure

Participants signed up for one of eight focus groups scheduled in the evening on different days. Kreuger and Casey [28] suggest between five and eight participants is optimal to gain a variety of perspectives while enabling a coherent and managed discussion. Due to absences, numbers in the eight focus groups varied from two to seven. The discussions were audio-recorded and lasted between 50 and 112 min (average 75 min), dependent on the number of participants attending. Each of the eight focus groups followed the same procedure and was directed by a discussion guide. As an introduction, the purpose of the focus group discussion was given, and then it was explained that everyone’s opinions were valued including those critical of the programme and participants were asked to be respectful of each other’s opinions. Following this, a series of key questions prompted group discussion to elicit opinions about the components of RuFIT-NZ that the men found useful/helpful and those that they found not so useful/helpful and how those factors helped or hindered their engagement with, and experiences of, the programme. The discussion concluded with the question, ‘what was the most important thing that kept you involved in RuFIT-NZ?’.

### 2.5. Analysis

Audio recordings were transcribed verbatim and this written text was the focus of the analysis. This text was subject to inductive thematic analysis as outlined by Braun and Clarke [31,32]. Thematic analysis is an analytical method used to systematically identify and report repeated patterns (themes) of shared meaning and experiences across a data set and can be applied within different theoretical frameworks [31]. A theme represents a patterned response or meaning with the data set that captures something of importance with respect to the research question. It was inductive to the extent that the analysis was data driven rather than trying to fit it into a pre-existing framework or theory. No analysis software was used in the analysis process. The data analysis procedure progressed through six phases as described by Braun and Clarke [31]. (1) To become familiar with the data, the transcripts were read thoroughly at least twice on top of checking the transcript for accuracy while listening to the audio recording. Initial ideas with respect to coding were written. (2) To generate initial codes, raw data quotes that related to the components of the programme that the men found or did not find helpful along with any explanations were identified and the idea within that quote was briefly described (i.e., given a code). Care was taken to ensure the quote also contained the context for the quote. (3) These quotes and codes were analysed for a patterned response (theme) and a first list of themes that seemed to represent the data was generated. (4) These themes were refined to ensure the themes reflected the meanings evident in the data set and that separate themes had distinct meanings. (5) Each theme was named to capture the essence of what each theme (and its sub-themes) represented. (6) Participant quotes were chosen for inclusion in the final report to illustrate the themes and to give voice to the participants in such a way that convinces the reader of the merit and validity of the results.

A relativist approach was adopted to evaluate the rigour of the work. Contextually situated criteria were drawn from contingent ever-changing list of characteristics traits that provide judgement on the quality of the work [33,34]. Here, the following criteria were included: (1) the worthiness of the topic, (2) its significant contribution, the resonance by evoking a sense of what it was like to be a participant in the RuFIT-NZ programme and meaningful coherence such that the methods and results achieved the stated purpose [35]. Rigour was enhanced through the use of a ‘critical friend’, where the first author explained their interpretation of the data themes to the other authors who offered critical feedback to challenge and develop the interpretation, resulting in a final, defensible thematic structure [36].

## 3. Results

The thematic analysis was driven by the question, ‘what factors inherent within the RuFIT-NZ programme influenced the men’s experiences of, and engagement with, the programme?’. Five themes characterised the data, (1) a group of like-minded men all in the same boat, (2) the sense that they were a team, (3) the motivational coach, (4) the education sessions, and (5) the involvement of the rugby franchise. Table 1 presents the five themes, along with the sub-themes and codes derived from the analysis.

### 3.1. A Group of Like-Minded Men All in the Same Boat

Participants had common characteristics, they were overweight and lacking in fitness, were in the same life situation juggling work and family life, and had a common interest in rugby. They also shared similar reasons and motivations for joining the programme, for example, to be more active and become fitter, to lose weight, to improve their health, to be a role model for their kids. Consequently, the men described themselves as ‘like-minded’, ‘all in the same boat’ and ‘there for the same reasons’ and this was an important factor for engagement with the programme. As participant 10 explained, ‘for me it [what kept him involved] was doing it with like-minded men. People that didn’t care about size, didn’t care about people’s fitness ability, we were all there supporting each other to achieve our own goal but as a group’. Similarly, Participant 52 added, ‘we all had the same goals, you know some were lifestyle, some was weight, some was fitness you know, but we all wanted to get lighter and fitter, you know in a nutshell … having a common bond like that brought us together quite well’. These common group characteristics meant that the men did not feel out of place or self-conscious, but that they ‘were meant to be there and it was your gym’ (Participant 1). The group-based, and male-only nature of the programme drew the men to the programme in the first place, it was described as being ‘much more inviting than trying to struggle all by yourself all the time’ (Participant 24). It also provided the men with a sense of responsibility and accountability to achieve their weight loss or fitness goals.

The results of this theme and its sub-themes suggest that these common characteristics, motivations, and life situations that the men brought with them to the RuFIT-NZ programme enhanced their engagement with it.

### 3.2. We Were a Team

The team environment that was created was described as an integral part of the RuFIT-NZ experience. The men described themselves as being ‘part of a team’, that there was a ‘team environment’ and they ‘were one tight unit’. For others the camaraderie that came with being in that team environment provided the motivation to turn up each week, ‘the camaraderie, you know … it was the guys and we bonded, we bonded really tightly and it was actually you went there cause you wanna be with the guys and you did it because the guys were doing it’ (Participant 45).

The support and motivation that the men provided to each other created the sense of being a team and was a key element of the programme’s success. The support manifested through the men helping each other through their workouts when they were struggling:

We were all supportive of each other and it wasn’t just about yourself, it was about making sure your mate got through as well. And in those teams… you were pushing the other person to make sure you’re, you know, not just to finish yourself but it was getting the other person through as well

(Participant 24)

This support meant the men did not feel like they had to ‘slog it out by yourself’ (Participant 34) and that someone was looking out for them:

A lot of the guys [were] concerned you know, if you didn’t show up, if you weren’t there the week before, then they would ask ‘are you alright?’, you know, make sure you’re alright so that was a big bonus. Makes you want to commit yourself even more to these guys cause they’re loyal to you, so you know, they’re not just there for themselves, they’re there for you as well

(Participant 23)

The motivating force of the team was illustrated through the men pushing each other to work hard during the training sessions, providing an element of competition, not against team mates but personally, to do their best to benefit the team, and being inspired by others’ achievements. For example, ‘there’s older men than me but I tend to be a lot more unfit than they are, so you know, to have them leading from the front too is sort of, just trying to keep up with them, is inspirational’ (Participant 23). Participant 40 summed up nicely:

What kept me going was the team environment…because you go there, the team is there, you were acting as a team helping each other out as a team, you know, cheering each other on as a team so you wanted to be part of that environment

(Participant 40)

Like a sport team, the men created informal values and expectations with respect to behaviour during the sessions based on being honest, non-judgemental, and following the team rules. For example, the reinforcement of team rules (e.g., wearing the RuFIT team t-shirt) created a sense of team responsibility. The honest conversations led others to speak freely in the group discussions, and there was no judgement:

I think from the very start when people started bringing their ideas or things … no-one ever got shut down, no-one ever got criticised for bringing up an idea. It was a very positive environment where everyone got supported and everyone’s opinions were valued and treasured

(Participant 48)

Yet alongside the seriousness of the conversations there was an important element of banter, humour and laughs; ‘we would have a bit of banter, but it was all a good part of it’ (Participant 15).

In a critique of the programme, the men felt that the maintenance of the team environment and social support network following the 12-week programme was missing and there was an abrupt end to the programme without a succession plan. They felt that they were not ‘ready for total independence’ (Participant 45) and still needed something to provide them with accountability for their behaviours.

### 3.3. The Motivational Coach

The RuFIT-NZ coach was integral to the men’s experience of the programme because of his personality characteristics and interpersonal skills, and the way he structured individualised training sessions. He was described as a role model, brilliant motivator, down to earth, enthusiastic, energetic, positive, encouraging, supportive and personable. For example, Participant 52 described him as a, ‘very unifying sort of person, he’s very motivating but also very down to earth, he knew where you were coming from sort of thing and it felt like he was actually invested into your best interests’. The trainer was integral in helping create the team atmosphere by modelling the same behaviours as the men, getting involved in the trainings, creating a comfortable and fun environment. The men appreciated his abilities to structure group-based training sessions that were varied, challenging and adapted to ensure a sense of achievement was experienced irrespective of ability. This was captured by Participant 20:

It [exercise sessions] was a killer but I did it, I was able to cope with it and I was like, this is brilliant, you know I’m finally being able to accomplish something physically which I haven’t been able to do for years and years and years. And you know that really brought my confidence up to say, ‘hey I can actually do this’ and I know, even though I was gasping for air and that sort of thing, I really enjoyed it.

This was important to the men because they still felt involved and part of the team. Clearly, the choice of person to deliver the programme is important to elicit participant engagement.

### 3.4. The Education Sessions

The education sessions were valuable to the men because they were based around messages emphasising small, realistic behaviour changes, they were gaining knowledge, and the methods used by the speakers to deliver the sessions were engaging. The men appreciated that the focus of the education sessions were on making small, realistic behaviour changes. For example, ‘it was sensible as well. It wasn’t you need to change everything right now, it’s kinda incremental changes and the right kind of thing you can do’ (Participant 47) and ‘they [speakers] were saying things that I didn’t wanna hear but I had to start listening you know and it was about small changes make the biggest difference, not big changes, they’re very realistic’ (Participant 45). For many the information being presented was not necessarily new to them, but they felt it was great to have it reinforced again and to get knowledgeable answers to questions straight away; ‘the education side of it was a big motivational thing too, you’re learning, reinforcing things that you should do. Even though you know it, but having a professional reinforce that was really good’ (Participant 7). One man commented that having professionals come in, ‘smashing that “bro” science, that you know, that’s been around forever, that’s was a biggie’ (Participant 9). The men also commented on how, during the sessions, they learned from each other; ‘yeah cause most people would share what they’d learnt, you know and what was working for them… it’s good to hear it’s working in someone’s life who’s you know got the same goals as you’ (Participant 48).

Registered dieticians delivered the sessions related to nutrition and the men specifically commented that these professionals passed on information in ways they could understand, and remember, in the context of their situation. They were also perceived as supportive and non-judgemental:

the attitude from them [speakers] was all to try and be as helpful as they possibly could and they were honoured to talk to us which made me feel a bit strange cause I’m honoured to listen to them. But yeah they looked at us as a group who wanted to make some changes and they were doing everything they could do to help us

(Participant 26)

This theme reinforces that the structure and content of the education sessions were appropriate and valued, and contributed to the success of the programme.

### 3.5. The Involvement of the Rugby Franchise

The men discussed the rugby franchises involvement in RuFIT-NZ as creating initial interest in signing up for the programme and an element of commitment to stick to the programme. One man commented, ‘yeah that’s a winner [rugby franchise involvement], it has to be, cause I wouldn’t have been interested otherwise. That’s what drew me to the ad in the newspaper’ (Participants 26). It also differentiated it from other lifestyle change programmes:

the involvement with the word ‘The Highlanders’ I think it made you sort of go for it, rather than just another programme in the paper that says they need volunteers to lose weight and all this stuff. I think to know that you’re going to be doing something at their gym. I think it was that that probably spurred me on

(Participant 39)

Participants spoke about being able to exercise in, and use the franchise’s facilities as ‘all part of the attraction’ (Participant 23), and that it provided the men with a home and a reminder of the enjoyment they got from their rugby days. They recognised that it was a privilege to use the facilities and having those, as well as franchise staff giving their time to speak to the men, motivated them to stick to the programme. Finally, they appreciated the support and recognition they obtained from the franchise staff for all the hard work they had put into the programme. They believed the rugby franchise was not just in it for the publicity but rather that they respected their fans.

In suggesting changes to the programme, the men expressed a desire for greater franchise involvement, for example, players dropping into the training sessions. It was also suggested that after the 12-week programme, the franchise could ‘own’ the programme and keep reinforcing it through for example, special invitations to games for RuFIT-NZ participants.

## 4. Discussion

From a health-care perspective, this evaluation of men’s experiences of the pilot RuFIT-NZ programme provides important information on the factors, or active ingredients, that should be inherent within a men’s healthy lifestyle programme to create engagement. Our results illustrated that engagement with the programme was heavily influenced by: (1) factors brought to the programme by the men who enrolled: their life situations, motivations and behaviours, and how those factors supported the creation of a team atmosphere; (2) the supportive, motivating and skilful coach who delivered the programme; and (3) factors inherent to the design and content of the programme: the knowledge gained from the education sessions and its situation within professional sport. Although the results may appear similar to those from evaluations of other ‘Fans in Training’ style lifestyle programmes [17,18,19,20,21], these programmes are all conducted slightly differently (for example, RuFIT-NZ used dieticians to deliver nutrition content, strength and conditioning coaches to deliver the main programme content), hence there is a need to understand participant experiences of each. Our results also add to the literature by providing a New Zealand and rugby union perspective and adding weight to the importance of the characteristics of the coach delivering the programme, the communication and presentation skills of those delivering the education sessions, and the efforts that should be encouraged to create a team-based environment. Interestingly, the importance of the franchise involvement for continued engagement did not come across as strongly as in previous research e.g., [17,21].

The finding that the men felt understood, supported and not self-conscious by being in a group with men with similar life situations (such as juggling work, and family commitments), physical characteristics (including being overweight and lacking fitness) and desire to improve their health, fitness and weight, is common to similar male-only lifestyle programmes [16,17,20]. Indeed, recognising the motivational force of peer-support from men with whom they can identify is one of the guiding philosophies of the RUFIT-NZ (and other ‘Fans in Training’) programmes. As other authors have suggested, this builds common connections between the men and they do not feel out of place, which provides the base from which behaviour change can develop [37]. Indeed, the results also showed that the men felt they were a team providing support and motivation to each other. They were not simply a group of individuals enrolled in the programme. This team element and the giving and receiving of social support was also reported in other professional sport-based healthy lifestyle interventions [16,17,20].

These findings may suggest that the men developed a social identity as a member of the RuFIT-NZ group, which could provide the mechanism to explain why these factors were stated as being integral to their continued engagement with the programme. The social identity approach [38,39] recognises that as well as having a personal identity, people can come to define themselves as a member of a particular group, creating a social identity. People with similar demographic and physical features, as well as similar beliefs and attitudes, like those men in the study, are common markers of groups with a shared identity [40]. Having a social identity is proposed to lead people to behave in ways that others in the group behave in if that identity is salient to them at that particular time, e.g., while they are participating in the RuFIT programme [39]. Furthermore, Haslem et al. [41] explain that when people have a salient social identity they are more likely to give, and be the receiver of social support from those whom they share a social identity, and be more likely to make use of the support. This may be especially relevant for Māori men in New Zealand who seem to be particularly drawn to programmes that play on their collective orientation to provide peer encouragement, accountability and a sense of obligation [42]. Research in the health behaviour context has shown that a salient social identity is associated with engaging in healthy lifestyle behaviours e.g., [43,44] and social identification-building interventions have a moderate to strong positive effect on health outcomes [45]. These conclusions remain speculative, and as identified by Steffens et al. [45] in their systematic review, future research should evaluate social identity as mechanism of action in group-based behaviour change interventions.

Another important element for promoting behaviour change in group-based interventions is the successful development of positive group dynamics where individuals are a collaborative group working towards common goals [37]. Many of the processes that create positive group dynamics [37] were evident in the men’s experiences reported here. Specifically, working toward individual goals that were common across the group (e.g., getting healthy, improving fitness, and losing weight), identifying as a group and having a sense of group cohesion and group norms around acceptable behaviour (e.g., having team rules). Furthermore, the interpersonal change processes that are fostered as a result of the group dynamics [37] were evident. Specifically, sharing experiences and learning from one another during the education and exercise sessions, being accountable to the group (both in behaviours and in giving 100% effort to the exercise sessions), and co-operation in helping each other.

The importance of the coach for creating engagement suggests that selecting a person with supportive and motivating professional, and interpersonal skills is paramount for the creation of the team atmosphere and in facilitating group interactions [16,37]. Unlike other professional sport based lifestyle interventions, the RuFIT-NZ coaches did not go through a rigorous training programme, we relied on an introduction to the philosophies of the programme along with their previous experiences and professional qualifications in strength and conditioning and personal training to ensure they were equipped to deliver the programme. Although this proved successful here, the lack of training was recognised as a potential limitation in the programme [6] and so in future, as well as ensuring the coaches have the characteristics outlined here, ensuring the coaches have standardised training to deliver the programme may enhance its effectiveness. This may also include adding aspects of identity leadership to enhance the creation of a social identity between group members [46].

The content and delivery of the education sessions were valued by participants. Similar to participants in FFIT [17] and Hockey FIT [19] the men found the sessions beneficial to reinforce old and provide new knowledge. Similar to the men in Hat Trick [20], they appreciated the realistic messages to make small, manageable behaviour changes. These elements were specifically built into the RuFIT-NZ programme given previous evidence that a small change approach can promote significant behaviour change and weight loss [47], these results provide further evidence that this approach is appreciated by men. In contrast to other sport-based lifestyle programmes, we chose to have professional dieticians deliver the nutrition content. Dieticians are perceived as one of the most trustworthy sources of nutrition information [48] and our results show that the professional delivery of the nutrition information was valued. Nonetheless, we also realise that from a health-care perspective this can limit the ease of future delivery of the programme outside the research content and add to the costs.

The programme’s situation within a professional rugby franchise provided a ‘hook’ for participants to sign up for the programme and differentiated it from other weight management programmes. However, the involvement of the franchise did not come across as being as important to the men’s engagement in RuFIT-NZ as it has in other sport-based lifestyle programmes, e.g., Hat Trick [20], FFIT [17] and Premier League Health [49]. In the Hat Trick programme, the resources were specifically tailored with hockey-related terminology, metaphors and imagery, which enhanced the connection to the sport and team. In FFIT, attending the programme at the football club was described as a ‘powerful draw’ and similar to our results the men felt privileged to use the club facilities. Here we provided rugby franchise branded t-shirts and programme materials, and while we presumed that the coaches would naturally include rugby-related terms in the programme delivery, there were no specific rugby-related terms or pictures in the participant manual. Consequently, it may be that the content and delivery of the programme did not do enough to cultivate the connection to the rugby franchise. Alternatively, it may be that some of the men in RuFIT-NZ were not ‘die hard’ rugby fans and so the setting was not as important to them, and it was other aspects of the programme (e.g., it being a male-only lifestyle intervention, or the opportunity to improve their health) that prompted participation and continued involvement in the study. However, when asked how the programme could be improved, greater rugby franchise involvement was suggested. This remains a logistical issue in terms of what the clubs are able and willing to provide in support of the programme and may not be an issue if the clubs themselves were running the programmes independently.

Despite asking the men to talk about the elements of the programme they felt both helped and hindered their engagement of the programme, the evidence provided was largely positive. In providing suggestions for future changes to the programme, comments focused on adding to what already existed, for example, greater rugby franchise involvement (e.g., players dropping into the training sessions), more exercise ‘homework’ on top of the walking programme, strengthening the goal-setting elements and changes to the participant booklet they were given. A more extensive critique of the programme may have emerged had we conducted individual interviews rather than focus groups. While we endeavoured to create an atmosphere where the men could share all their experiences, and indeed some suggestions for improvement were provided, some men may have been reluctant to voice any negative concerns in the group setting, particularly also given that the facilitator was involved in running the intervention. An additional limitation was that only men from one site of the pilot intervention were interviewed, a decision made for logistical reasons. Consequently, the experiences of the men from the Auckland Blues arm of the intervention may have provided additional detail. It should also be recognised that these results come from a group of men (the majority of whom were married, educated and earning above median income levels for New Zealand) who were attracted to participating in a sport- and group-based healthy lifestyle programme and the results should be interpreted with this in mind. Furthermore, it is likely that the men who sign up for these programmes have personalities more suited to group interaction and the creation of positive group dynamics than those who do not. Therefore, although these programmes are certainly attracting at-risk men [9], research has yet to establish if certain groups of men are not participating.

## 5. Conclusions

Understanding the experiences of men who participate in men-only healthy lifestyle interventions is important to inform their continued development and improvement so as to influence the health of men. These results from the pilot study of the RuFIT-NZ programme demonstrated that for New Zealand men, the key factors to its success were the non-judgemental, supportive and motivational environment created by the men themselves, and the personnel delivering the programme, as well as the new knowledge they gained to implement changes to their own lifestyles. These findings will be used to strengthen the design and delivery of the RuFIT-NZ programme to be evaluated in a definitive trial to examine its efficacy over a 12-month period. These findings are also relevant for informing development of other healthy lifestyle interventions designed specifically to engage men.

## Figures and Tables

**Table 1:** Thematic analysis of factors explaining engagement with the Rugby Fans in Training—New Zealand programme (RuFIT-NZ) pilot trial.

Initial Code	Sub-Theme	Theme
Like-minded men Same reasons for joining RuFIT Same life situation		A group of like-minded men all in the same boat
Were a Team Camaraderie (friendship)	Team environment	We were a team
Group supported each other Group motivated each other	Team support and motivation	
Honesty Non-Judgemental Team Rules	Team values and expectations	
Role model Motivating, supportive and encouraging Knowledge Role in creating team atmosphere	Coach personality and interpersonal skills	The motivational coach
Understood everyone’s physical abilities Adapted exercises to meet individual needs	Individualised training	
Training intensity Variety of exercise training Exercises were challenging & created a sense of achievement Group-based training	Structure of each training session	
	Realistic messages	The education sessions
Reinforced current knowledge Learning new knowledge Learning from others in the group	Knowledge creation	
Techniques to deliver information Information participant-tailored Knowledgeable Supportive and non-judgemental	Skilled & knowledgeable speakers	
Created interest for the programme Privilege to be in Franchise facilities Gym environment was motivating Connection to rugby Franchise staff involvement	Created interest for, and maintained commitment to RuFIT	Rugby franchise involvement
	Recognition/indirect support for participant achievements	

## Data Availability

The data that support the findings of this study are not publicly available due to ethical restrictions on sharing qualitative data. The full focus group guide can be provided upon request.
